# Carvacrol Protects against Hepatic Steatosis in Mice Fed a High-Fat Diet by Enhancing SIRT1-AMPK Signaling

**DOI:** 10.1155/2013/290104

**Published:** 2013-02-20

**Authors:** Eunkyung Kim, Youngshim Choi, Jihee Jang, Taesun Park

**Affiliations:** Department of Food and Nutrition, Yonsei University, 50 Yonsei-ro, Seodaemun-gu, Seoul 120-749, Republic of Korea

## Abstract

We investigated the protective effect of carvacrol against high-fat-diet-induced hepatic steatosis in mice and the potential underlying molecular mechanisms. Mice were fed a normal diet, high-fat diet, or carvacrol-supplemented high-fat diet for 10 weeks. Compared to mice fed the high-fat diet, those fed the carvacrol-supplemented diet showed significantly lower hepatic lipid levels and reduced plasma activities of alanine aminotransferase and aspartate aminotransferase and plasma concentrations of monocyte chemoattractant protein 1 and tumor necrosis factor **α**. Carvacrol decreased the expression of LXR**α**, SREBP1c, FAS, leptin, and CD36 genes and phosphorylation of S6 kinase 1 protein involved in lipogenesis, whereas it increased the expression of SIRT1 and CPT1 genes and phosphorylation of liver kinase B1, AMP-activated protein kinase, and acetyl-CoA carboxylase proteins involved in fatty acid oxidation in the liver of mice fed the high-fat diet. These results suggest that carvacrol prevents HFD-induced hepatic steatosis by activating SIRT1-AMPK signaling.

## 1. Introduction

Simple hepatic steatosis, once considered benign, is now being recognized as a condition that may lead to steatohepatitis (hepatic steatosis with inflammation), fibrosis, and ultimately cirrhosis. The risk factors associated with hepatic steatosis are varied and include diabetes mellitus [1], hypertension [2], and obesity [3]. Several studies suggest that excessive fat accumulation in the liver occurs due to increased hepatic *de novo* lipogenesis, impaired fatty acid oxidation, or export of triglycerides. Mounting evidence suggests that a high-fat diet (HFD) causes enhanced lipogenesis and impaired fatty acid oxidation by inhibiting AMP-activated protein kinase (AMPK) activation through Sirtuin 1 (SIRT1), leading to the development of hepatic steatosis.

The role of dietary cholesterol, with the subsequent increased hepatic esterification of cholesterol and its association to hepatic triglyceride accumulation, is a new paradigm for hepatic steatosis [4]. Cholesterol is accumulated in the liver under excess dietary cholesterol intake by disrupting the balance among steroid hormone synthesis, cholesterol uptake, and cholesterol efflux [5]. Accumulated cholesterol is esterified by acyl-coenzyme A:cholesterol acyltransferase (ACAT) in the liver, where some of it can be stored within hepatocytes in lipid droplets as cholesterol esters. When excess stored cholesterol ester molecules are present in the liver, the mobilization of hepatic triglyceride is limited and triglyceride secretion is reduced, resulting in the retention of neutral lipids as lipid droplets within the liver [4].

Carvacrol [isopropyl-ortho-cresol, C_6_H_3_(OH)(C_3_H_7_)] is a predominant monoterpene phenol which occurs in many essential oils of the family Labiatae including *Origanum*, *Satureja*, *Thymbra*, *Thymus*, and *Coridothymus* species [6]. Carvacrol is a food additive approved by the US Food and Drug Administration and is a legally registered flavoring and foodstuff in the Council of Europe (2000). It is reported that carvacrol appears to be slowly adsorbed into the rabbit intestine after oral administration [7]. After 22 h, about 30% of 1.5 g carvacrol was still in the gastrointestinal tract while 45% was absorbed into the intestines in rabbit [7]. Previous *in vitro* studies demonstrated positive effects of carvacrol on inflammation, cancer, and oxidants [8–10]. It was found to decrease cyclooxygenase-2 expression in human macrophage-like U937 cells [8], Bcl2/Bax ratio and poly(ADP-ribose) polymerase-1 cleavage in breast cancer cells [9], and lipid peroxidation induced by reactive free radicals [10]. Several rodent studies have shown that carvacrol provides protection against various pharmacological properties, including antidepressant [11], anxiolytic-like [12], antinociceptive [10], and hypotensive [13] activities. Furthermore, Aristatile et al. reported that carvacrol exerted a beneficial effect in hepatotoxicity through decreased activities of plasma alanine aminotransferase (ALT) and aspartate aminotransferase (AST) in D-galactosamine-induced hepatotoxic rats [6, 14]. Although a number of studies have been carried out to investigate the biochemical roles of carvacrol, the protective activity of carvacrol against hepatic steatosis has never been reported. Therefore, the main objective of this study was to investigate the protective effects of carvacrol against HFD-induced simple hepatic steatosis in mice and to study potential molecular mechanisms, focusing on the expression of genes involved in lipogenesis and fatty acid oxidation in the liver.

## 2. Experimental Procedures

### 2.1. Animal Studies

Male C57BL/6N mice (5 weeks old) were obtained from Orient Bio (Gyeonggi-do, South Korea) and maintained under 12 h light-dark cycles with free access to food and water. They were divided into 3 experimental diet groups (*n* = 8 per group): normal diet (ND), HFD, and carvacrol-supplemented diet (CSD). The ND was a purified diet based on the AIN-76 rodent diet composition. The HFD was identical to the ND, except that 200 g fat/kg (170 g lard plus 30 g corn oil) and 1% cholesterol were added to it. The CSD was identical to the HFD and contained 0.1% (w/w) carvacrol (Sigma, MO, USA). The experimental diets were given ad libitum for 10 weeks in the form of pellets. At the end of the experiment, all animals were anesthetized with ether, blood was collected in EDTA-coated tubes and centrifuged, and plasma was stored at −70°C. Livers were removed, weighed, and stored at −70°C. All mice were housed in the specific pathogen-free facility of the Yonsei University, Seoul, Korea. This study was approved by the Institutional Animal Care and Use Committee of Yonsei University.

### 2.2. Biochemical Analysis

Plasma activities of ALT and AST were measured using commercial kits (Bio-Clinical System, Gyeonggi-do, South Korea). Hepatic lipids were extracted from whole liver homogenates using a modified Folch extraction. Levels of triglycerides, free fatty acids, and cholesterol in hepatic lipid extracts were measured using commercial kits (Bio-Clinical System, Gyeonggi-do, South Korea). For measurement of hepatic cholesteryl esters, lipids were extracted from frozen liver tissues by thawing and homogenizing in chloroform (Sigma) : isopropanol (Sigma) : NP40 (Sigma) (7 : 11 : 0.1). The tissue homogenates were centrifuged (15,000 ×g, 10 min, 4°C) and the resulting supernatants (organic phase) were used for the cholesterol ester analysis. Total cholesterol and free cholesterol levels were measured using commercially available kits (ABCAM, Cambridge, UK). The level of cholesteryl esters was calculated by subtraction of the obtained values of free cholesterol from total cholesterol. Plasma levels of tumor necrosis factor-alpha (TNF*α*) and monocyte chemoattractant protein-1 (MCP1) were measured using ELISA kits (ID Labs, MA, USA).

### 2.3. Liver Histology

Liver sections were formalin fixed and paraffin embedded prior to sectioning. All sections were then stained with hematoxylin (Sigma) and eosin (Sigma), encoded, and assessed for steatosis and inflammation, by an expert liver pathologist blinded to the identity of the groups. The grade of steatosis was scored as 0 = no steatosis; 1 = minimal steatosis; 2 = slight steatosis; 3 = moderate steatosis; 4 = marked steatosis; 5 = severe steatosis. The grade of lobular inflammation was scored as 0 = no inflammatory foci; 1 = 1-2 inflammatory foci; 2 = 3-4 inflammatory foci; 3 = <4 inflammatory foci.

### 2.4. Hepatic Gene Expression Analysis

Total RNA was isolated from liver tissue by acid guanidinium thiocyanate-phenol chloroform extraction using Trizol reagent (Invitrogen, CA, USA). Four micrograms of total RNA were reverse transcribed using the Superscript II kit (Invitrogen, CA, USA) according to the manufacturer's recommendations. Primers used for polymerase chain reaction (PCR) are listed in Table 1. Taq DNA polymerase was used to amplify transcribed genes using a PCR program of a denaturation step of 10 min at 94°C, followed by 30 cycles of 30 s at 94°C, 30 s at 55°C, and 1 min at 72°C, then 10 min at 72°C, and terminated by an elongation step at 72°C for 10 min. PCR products were size fractionated on a 2% agarose gel and stained with ethidium bromide. 

### 2.5. Western Blot Analysis

Liver tissues of each mouse were homogenized at 4°C in an extraction buffer containing 100 mM Tris-HCl, pH 7.4, 5 mM EDTA, 50 mM NaCl, 50 mM sodium pyrophosphate, 50 mM NaF, 100 mM orthovanadate, 1% Triton X-100, 1 mM phenylmethanesulfonyl fluoride, 2 *μ*g/mL aprotinin, 1 *μ*g/mL pepstatin A, and 1 *μ*g/mL leupeptin. The tissue homogenates were centrifuged (1300 ×g, 20 min, 4°C) and the resulting supernatants (whole-tissue extracts) were used for western blot analysis. The total protein concentrations of the whole-tissue extracts were determined by Bradford assay (Bio-Rad, CA, USA). Protein samples were separated with 8% sodium dodecyl sulfate-polyacrylamide gel electrophoresis, transferred onto a nitrocellulose membrane (Amersham, Buckinghamshire, UK), and hybridized with primary antibodies (diluted 1 : 1000) overnight at 4°C. The membrane was then incubated with the appropriate secondary antibody and immunoreactive signals were detected using a chemiluminescent detection system (Amersham, Buckinghamshire, UK). The signals were quantified using the Quantity One analysis software (Bio-Rad, CA, USA). Antibodies to liver kinase B1 (LKB1), phospho-LKB (Ser428), AMP-activated protein kinase (AMPK), phospho-AMPK (Thr172), acetyl-CoA carboxylase (ACC), phospho-ACC (Ser79), S6 kinase 1 (S6K1), phospho-S6K1 (Thr389), interferon regulatory factor 3 (IRF3), phospho-IRF3 (Ser396), and *β*-catenin were purchased from Cell Signaling Technology (Cell Signaling Technology, MA, USA) and antibody to *β*-actin was obtained from Santa Cruz Biotechnology (Santa Cruz, CA, USA).

### 2.6. Statistical Analysis

The mean ± SEM of body weight gain, liver weight, and plasma and hepatic biochemistries was determined from 3 independent experiments. Reverse transcription (RT)-PCR and Western blot data were presented as mean ± SEM of at least 3 separate experiments. All of the analyses were performed using SPSS statistical software. Statistical analysis of results was performed using one-way analysis of variance (one-way ANOVA test), followed by Duncan's multiple-range tests. Statistical significance was set at *P* < 0.05.

## 3. Results

### 3.1. Carvacrol Reverses HFD-Induced Hepatic Steatosis

**Figure 1:**
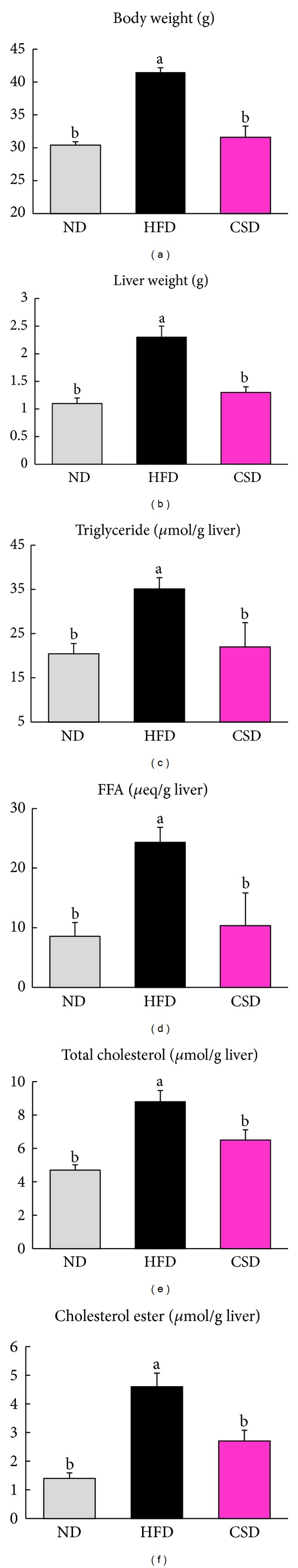
CSD mice are resistant to HFD-induced liver enlargement and hepatic lipid levels. (a) Final body weight of mice on ND, HFD, or CSD. (b) Weights of livers and (c–f) hepatic triglyceride, FFA, total cholesterol, and cholesterol ester levels. Data are mean ± SEM, *n* = 8. *P < 0.05.

**Figure 2:**
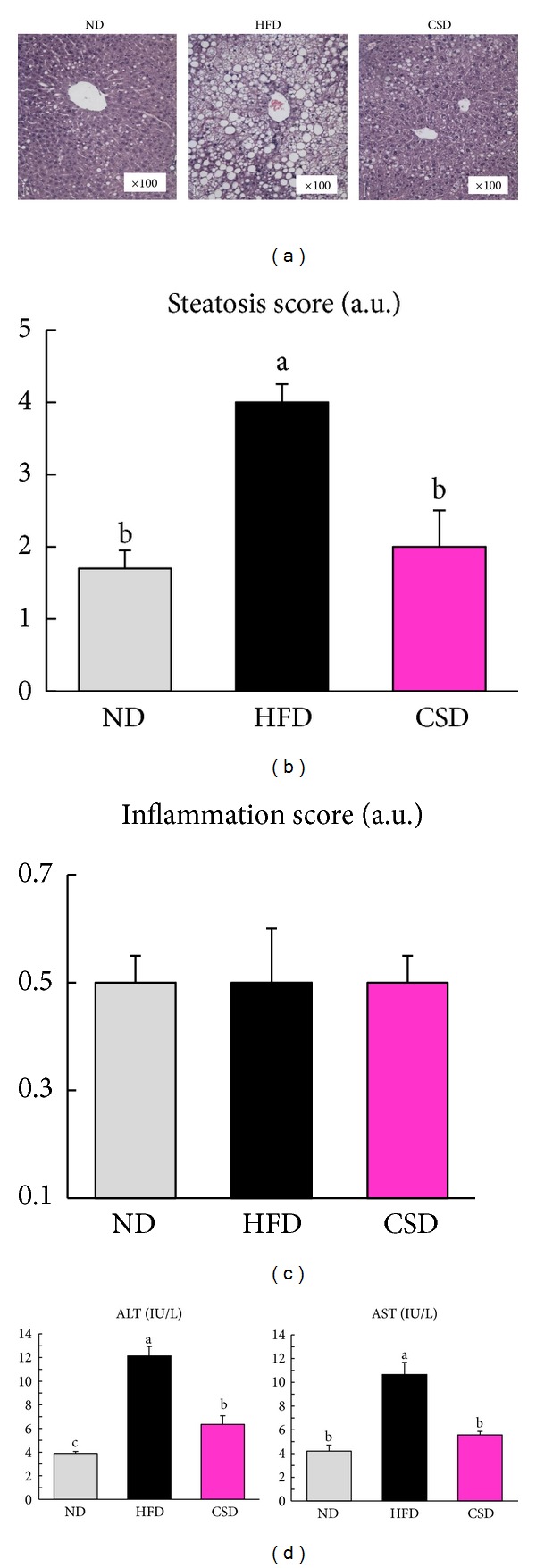
Carvacrol reduced hepatic lipid droplet and activities of ALT and AST. (a) Hematoxylin and eosin staining of representative liver section (magnification ×100). (b) Mean steatosis score. (c) Mean inflammation score. (d) Plasma ALT and AST activities. Data are mean ± SEM, *n* = 8. *P < 0.05.

At week 10, male mice fed the CSD displayed a significant reduction in final body weight compared with HFD-fed mice (Figure 1(a)). There was no difference in the food consumption among groups (data not shown). CSD-fed mice showed significant decreases in liver weight (43%, *P* < 0.05) compared to HFD-fed mice (Figure 1(b)). Hepatic triglycerides (37%, *P* < 0.05), free fatty acids (57%, *P* < 0.05), total cholesterol (26%, *P* < 0.05), and cholesteryl ester (41%, *P* < 0.05) levels were significantly higher in HFD-fed mice than in ND-fed mice, whereas CSD-fed mice were completely resistant to HFD-induced hepatic lipid accumulation (Figures 1(c)–1(f)). Histological sections of liver tissue from HFD-fed mice showed predominantly large lipid-filled vacuoles. Liver sections from CSD-fed mice revealed a reduction of lipid accumulation in the form of lipid droplets, or even small lipid droplets (Figure 2(a)). The hepatic steatosis scores in CSD-fed mice were significantly lower than scores in HFD-fed mice (Figure 2(b)). Evaluation of hepatic inflammation using hematoxylin and eosin liver staining revealed no significant differences between CSD- and HFD-fed mice (Figure 2(c)). As expected, plasma activities of ALT (47%, *P* < 0.05) and AST (47%, *P* < 0.05) were both substantially elevated by the HFD, and the CSD resulted in significant reductions in these plasma activities (Figure 2(d)).

### 3.2. Carvacrol Modulates the Expression of Genes Involved in Lipid Metabolism

**Figure 3:**
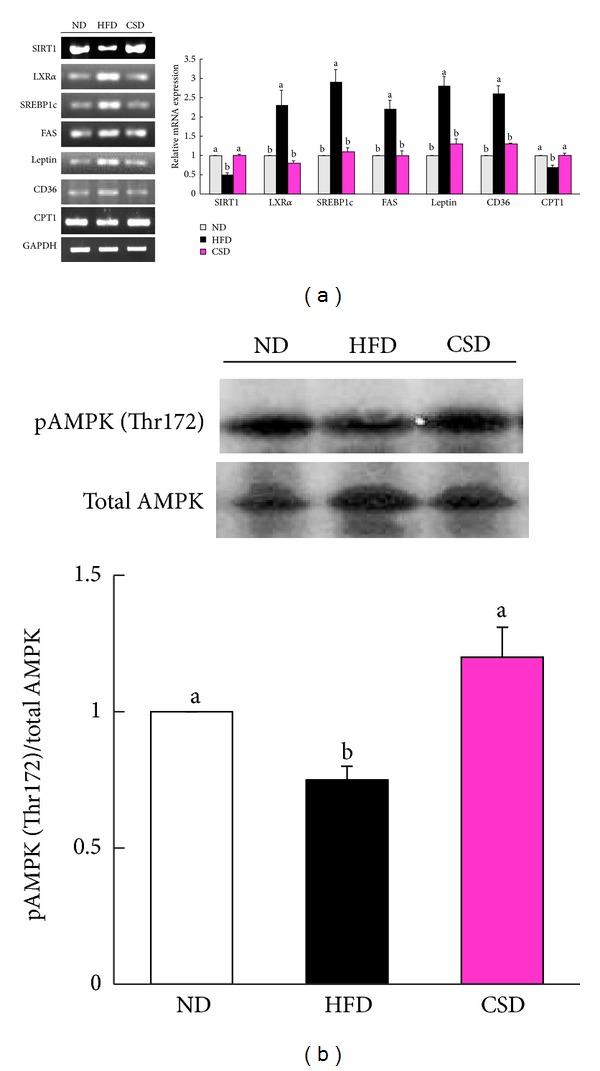
Enhanced hepatic SIRT1-AMPK signaling in CSD-fed mice. (a) Hepatic mRNA expression levels of SIRT1, LXR*α*, SREBP1c, FAS, leptin, CD36, and CPT1 normalized to GAPDH relative to ND-fed mice. (b) Upper, representative western blot of phospho- and total AMPK in livers of ND, HFD, and CSD-fed mice. Lower, densitometric analysis of AMPK phosphorylation expressed as change relative to each control band. Data are shown as the mean ± SEM, *n* = 8. *P < 0.05.

To gain insight into the protective mechanisms of carvacrol against hepatic steatosis in HFD-fed mice, we examined hepatic mRNA levels for genes involved in lipogenesis and fatty acid oxidation by RT-PCR analysis. SIRT1 and AMPK are key regulators of both lipogenesis and fatty acid oxidation in the liver. Expression of SIRT1 and phosphorylation of AMPK protein were significantly increased in the livers of CSD-fed mice compared with HFD-fed mice (Figures 3(a) and 3(b)). Hepatic mRNA levels of the lipogenic genes, including liver X receptor alpha (LXR*α*), sterol regulatory element binding transcription factor 1 (SREBP1c), fatty acid synthase (FAS), leptin, and CD36, were also significantly lower in CSD-fed mice than in HFD-fed mice (Figure 3(a)). In addition, expression of carnitine palmitoyltransferase 1 (CPT1), a reflection of mitochondrial *β*-fatty acid oxidation capacity, was significantly increased in the liver of CSD-fed mice compared with HFD-fed mice (Figure 3(a)).

**Figure 4:**
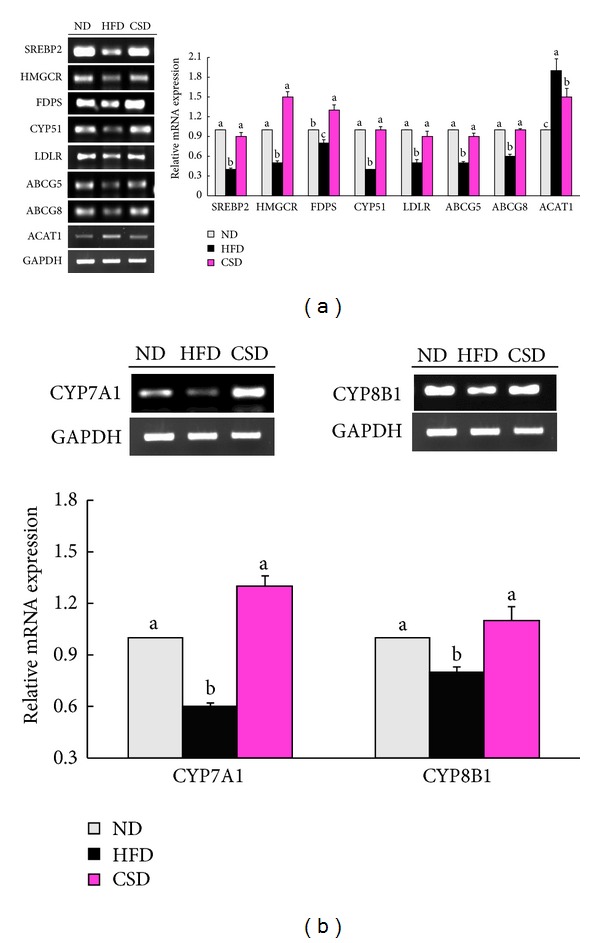
Enhanced hepatic cholesterol metabolism in CSD-fed mice. (a and b) Hepatic mRNA expression levels of SREBP2, HMGCR, FDPS, CYP51, LDLR, ABCG5, ABCG8, ACAT1, CYP7A1, and CYP8B1 normalized to GAPDH relative to ND-fed mice. Data are shown as the mean ± SEM, *n* = 8. *P < 0.05.

We also examined the effect of carvacrol on the expression of genes involved in cholesterol homeostasis in the liver. Hepatic mRNA levels of SREBP2 and its target gene LDLR, an important cholesterol influx transporter, were higher in CSD-fed mice than HFD-fed mice. CSD-fed mice had increased mRNA levels of genes involved in cholesterol synthesis, including 3-hydroxy-3-methylglutaryl-CoA reductase (HMGCR), Farnesyl diphosphate synthase (FDPS), and Cytochrome P450, family 51 (CYP51) in the liver compared with HFD-fed mice. Expression of ACAT1 was significantly decreased in the livers of CSD-fed mice compared with HFD-fed mice (Figure 4(a)). Expressions of ATP-binding cassette, subfamily G, members 5 and 8 (ABCG5, ABCG8) genes involved in cholesterol efflux, Cytochrome P450, family 7, subfamily A, polypeptide 1 (CYP7A1), and Cytochrome P450, family 8, subfamily A, polypeptide 1 (CYP8B1) genes involved in bile acid synthesis, were also higher in CSD-fed mice compared with HFD-fed mice (Figures 4(a) and 4(b)).

### 3.3. Carvacrol Inhibited the Expression of Genes Involved in Inflammation

**Figure 5:**
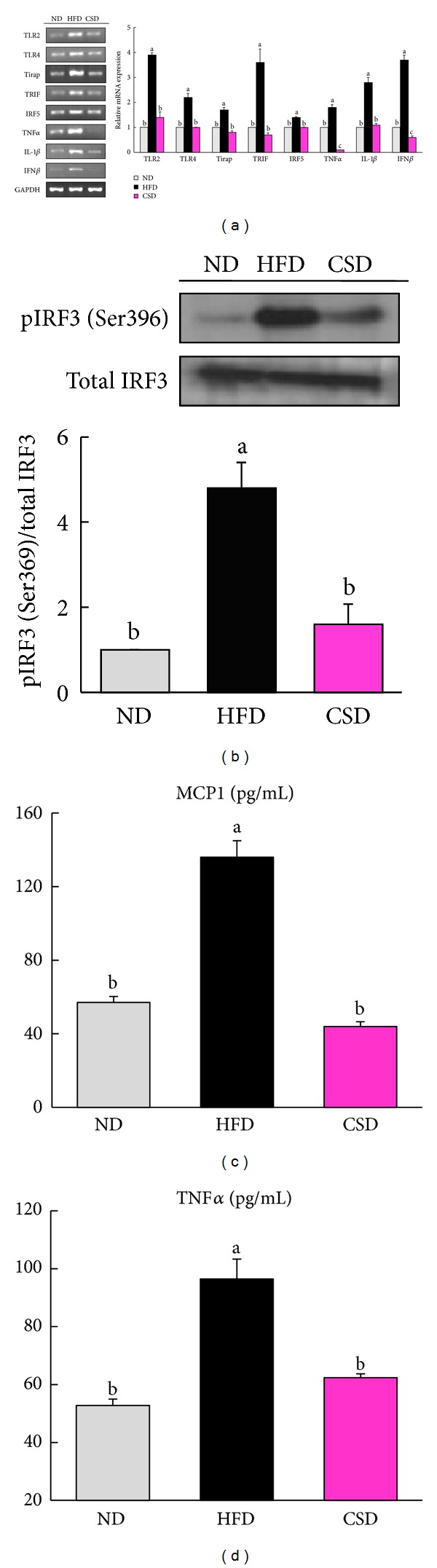
Reduced hepatic TLR2- and 4-mediated signaling and plasma levels of inflammatory markers in CSD-fed mice. (a) Hepatic mRNA expression levels of TLR2, TLR4 and related genes normalized to GAPDH expression relative to ND-fed mice. (b) Representative blots of hepatic IRF3 and phosphorylated IRF3 proteins in total liver extracts from the ND-, HFD-, and CSD-fed mice. Blots were quantified and the data are presented as the ratio of phosphorylated IRF3 to native protein, with values normalized to ND-fed mice. (c and d) Plasma MCP1 and TNF*α* levels. Values are means ± SEM from 8 animals, *P < 0.05.

In the livers of CSD-fed mice, levels of Toll-like receptors 2 and 4 (TLR2, TLR4) and their adaptor proteins (Toll-interleukin 1 receptor domain-containing adaptor protein (TIRAP) and TIR domain-containing adapter protein inducing interferon beta (TRIF)) were significantly reduced compared with their corresponding levels in HFD-fed mice (Figure 5(a)). Phosphorylation of interferon regulatory factor-3 (IRF3) protein, a key transcriptional factor in interferon beta (IFN*β*) induction, was decreased in the livers of CSD-fed mice compared to HFD-fed mice (Figure 5(b)). Significantly, higher levels of transcription factor interferon regulatory factor-5 (IRF5) and proinflammatory cytokines (interleukin [IL]-1*β*, IFN*β*, and TNF*α*) were also found in the livers of CSD-fed mice, as compared to those in HFD-fed mice (Figure 5(a)). Mice that received carvacrol showed significantly lower plasma concentrations of MCP1 (−67%) and TNF*α* (−35%) in comparison with the values for HFD control mice (Figures 5(c) and 5(d)). 

## 4. Discussion

The 0.1% carvacrol dosage (equivalent to 100 mg/kg body weight) given to mice in our study was chosen on the basis of previous reports. In these reports, D-galactosamine-induced hepatotoxic rats treated with carvacrol (80 mg/kg body weight) had significantly decreased plasma ALT and AST activities, as well as hepatic free fatty acid and cholesterol levels in comparison with saline-treated hepatotoxic rats [6, 14]. Mice treated with carvacrol (100 mg/kg body weight) showed reduced nociceptive behaviors induced by acetic acid as compared to vehicle-treated controls [10]. In our preliminary study, carvacrol supplemented to the HFD at 0.01, 0.05, and 0.1% levels for 28 days resulted in a dose-dependent reduction in the body weight of mice (data not shown). On the basis of these results, animals were fed 0.1% carvacrol for a longer period in the present study. Considering that the LD_50_ value for a single i.g. administration of carvacrol to rat was 810 mg/kg body weight in an acute toxicity study [15], the 0.1% carvacrol supplemented in the diet (equivalent to 100 mg/kg body weight) appears to have no harmful effect. The daily carvacrol intake of the mice in our study (100 mg/kg body weight) was equivalent to an intake of approximately 8.1 mg/kg human body weight (486 mg/60 kg person), when calculated on the basis of normalization to body surface area as recommended by Reagan-Shaw et al. and the US Food and Drug Administration (http://www.fda.gov/cder/cancer/animalframe.htm). The daily doses of commercial dietary supplements range from 9 to 288 mg carvacrol (0.15–4.8 mg/kg body weight) for a 60 kg human.

Several studies have demonstrated that the inactivation of SIRT1-AMPK signaling increases lipogenesis and represses rates of fatty acid oxidation in the livers of HFD-fed mice. Inactivation of SIRT1 leads to decreased deacetylation of Lys48 and possibly other key lysine residues on LKB1. This, in turn, inhibits LKB1 binding to STE20-related adaptor protein and mouse embryo scaffold protein, which inactivates its kinase activity and leads to the inhibition of AMPK phosphorylation [16]. Inactivation of AMPK through S6K1 activates LXR*α*, leading to the expression of target genes such as CD36, leptin, and FAS, which may contribute to increased fat accumulation in the liver. At the same time, inactivated AMPK increases ACC phosphorylation, subsequently decreasing the level of CPT1 in the liver. The consequence of this may be a decrease in fatty acid oxidation rates in the liver. In the present study, carvacrol reversed the HFD-induced upregulation of hepatic genes involved in lipogenesis (S6K1, LXR*α*, SREBP1c, FAS, leptin, and CD36) and HFD-induced downregulation of hepatic genes involved in fatty acid oxidation (SIRT1, AMPK, and CPT1). Accordingly, changes in expression of genes involved in lipogenesis and fatty acid oxidation may have contributed to the reduction of hepatic triglyceride and free fatty acid concentrations in CSD-fed mice.

The cells that internalize exogenous cholesterol repress endogenous cholesterol biosynthesis and LDLR expression in response to cholesterol loading. The hepatic cholesterol depletion was associated with compensatory mechanisms aimed at increasing hepatic cholesterol, including upregulation of HMGCR and LDLR [5]. In the present study, carvacrol decreased the HFD-induced increase in hepatic cholesterol concentrations and, simultaneously, increased the mRNA expression of hepatic HMGCR and LDLR. The elevated HMGCR and LDLR mRNA levels may be secondary to the reduced hepatic cholesterol concentrations induced by carvacrol supplementation. Another important protective mechanism against hepatic cholesterol accumulation is cellular efflux of cholesterol and bile acid biosynthesis [17, 18]. In the present study, carvacrol reversed the HFD-induced downregulation of CYP7A1 and CYP8B1 genes involved in bile acid biosynthesis and ABCG5 and ABCG8 genes involved in cholesterol efflux in the liver of mice. Therefore, the increased expression of these genes might contribute to the lower cholesterol concentration in the liver of CSD-fed mice.

The present study showed that carvacrol reversed the HFD-induced increase in free cholesterol and cholesterol ester concentrations in the liver of mice. In the hepatocyte, cholesterol exists as free cholesterol and as cholesterol esters [19]. It has been suggested that an increase in the intrahepatic free cholesterol concentration is rapidly balanced by an increase in the rate of cholesterol esterification to prevent excess cellular free cholesterol accumulation [20]. A recent study showed that the increased cholesterol ester in lipid droplets could limit the hydrolysis of triglycerides and decrease hepatic triglyceride secretion out of cells, leading to hepatic steatosis in the liver of mice fed a low-fat diet containing cholesterol [4]. Therefore, the protective action of carvacrol against hepatic steatosis might involve not only enhanced SIRT1-AMPK signaling, but also a decreased concentration of cholesterol ester.

TLRs play an important role in the innate immune system by activating inflammatory pathways in response to microbial agents [21]. TLR2 and 4 initiate shared and distinct signaling pathways by recruiting various combinations of the Toll-interleukin 1 receptor domain-containing adaptor proteins MyD88, TIRAP (Mal), TRIF, and TRAM. These signaling pathways activate the transcription factor IRF5, leading to the production of inflammatory cytokines. TLR4 also activates the transcription factor IRF3, leading to the production of type I interferons [21, 22]. TLR2- and 4-mediated signaling has emerged as a major mechanism involved in regulating inflammatory responses in mouse models of HFD-induced steatosis [23, 24]. Although no infiltration of inflammatory cells was detected in the livers of CSD- and HFD-fed mice, the expressions of proinflammatory cytokines (TNF*α*, IFN*α*, and IL-6) and their upstream signaling molecules (TLR2/4, TIRAP, TRIF, TRAF6, and IRF5) were decreased in CSD-fed mice compared with HFD-fed mice. The HFD-induced elevations in plasma TNF*α* and MCP1 concentrations were also significantly reversed by carvacrol supplementation. These findings support the recent *in vivo* studies on the anti-inflammatory activity of carvacrol. Guimaraes et al. [25] revealed that carvacrol significantly decreased TNF-*α* levels in pleural lavage and suppressed the recruitment of leukocytes without altering the morphological profile of these cells. Carvacrol has been reported to cause anti-inflammatory effects by reducing the production of inflammatory mediators, such as IL-1*β* and prostanoids, possibly through the induction of IL-10 release in a classical inflammation mouse model [26].

Our results are in accordance with previous studies showing that at the early stage of obesity induced by the HFD, the expression levels of the proinflammatory cytokines were increased prior to macrophage infiltration [23, 27]. HFD-induced fatty liver diseases can progress from simple steatosis to nonalcoholic steatohepatitis (NASH, fatty changes with inflammation and hepatocellular injury or fibrosis). It is well established that mice fed the HFD for 10 weeks showed simple steatosis with the absence of necrosis or signs of inflammation [28]. Although NASH did not develop in our 10-week experiment, upregulation of proinflammatory cytokines and profibrotic genes could have facilitated the deterioration of steatosis to NASH if the experiment had been conducted for a longer duration. Accordingly, the carvacrol-mediated reduction in the expressions of proinflammatory cytokines and plasma MCP1 and TNF*α* concentrations in the livers of HFD-fed mice may contribute to decreased infiltration of macrophage into the liver. 

**Figure 6:**
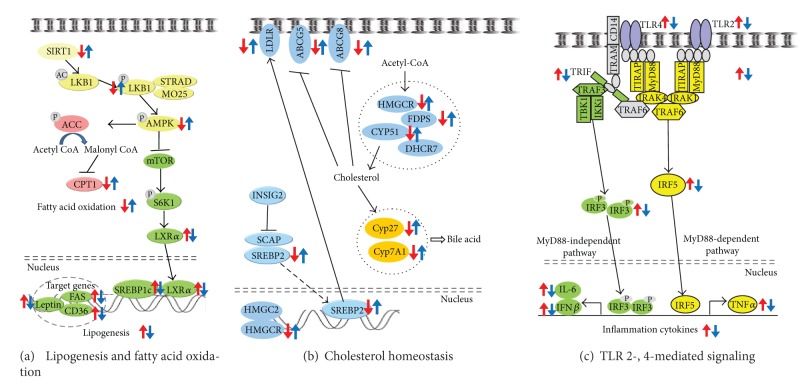
(a) Proposed mechanism for the protective effects of carvacrol against hepatic steatosis in mice. Carvacrol decreased the expression of genes and phosphorylation of protein involved in lipogenesis, whereas it increased the expression of genes and phosphorylation of proteins involved in fatty acid oxidation in the livers of HFD-fed mice. (b) Schematic overview of cholesterol homeostasis and the effects of carvacrol in the livers of HFD-fed mice. Carvacrol lowers cholesterol content by reversing the HFD-induced downregulation of genes involved in cholesterol homeostasis. (c) Schematic overview of the genes regulated by carvacrol in TLR2- and 4-signaling pathway.

In conclusion, carvacrol supplementation (0.1%) suppressed the HFD-induced increases in liver weight, hepatic lipid levels, plasma activities of ALT and AST, and the steatosis score in mice. The protective action of carvacrol against HFD-induced hepatic steatosis in mice appears to be mediated through the downregulation of genes involved in lipogenesis and upregulation of genes involved in fatty acid oxidation via SIRT1-AMPK signaling. Furthermore, carvacrol supplementation also provoked decreased expression of genes involved in TLR-mediated signaling cascades and reduced concentrations of plasma TNF*α* and MCP1, which may diminish hepatic inflammatory stress (Figure 6).

## Figures and Tables

**Table 1:** Primer sequences and PCR conditions.

Gene description	Primers	Sequences (5′ → 3′)	Annealing temperature (°C)	PCR product (bp)
Sirtuin1 (SIRT1)	F	CAGAACCACCAAAGCGGAAA	55	693
	R	GGCACTTCATGGGGTATAGA		
Liver X receptor (LXR*α*)	F	TCCTACACGAGGATCAAGCG	55	119
	R	AGTCGCAATGCAAACACCTG		
SREBP1c (SREBP1c)	F	TTGTGGAGCTCAAAGACCTG	55	94
	R	TGCAAGAAGCGGATGTAGTC		
CD36 antigen (CD36)	F	ATGACGTGGCAAAGAACAGC	55	160
	R	GAAGGCTCAAAGATGGCTCC		
Lipoprotein lipase (leptin)	F	CTCCAAGGTTGTCCAGGGTT	55	143
	R	AAAACTCCCCACAGAATGGG		
Fatty acid synthase (FAS)	F	AGGGGTCGACCTGGTCCTCA	65	132
	R	GCCATGCCCAGAGGGTGGTT		
Carnitine palmitoyltransferase I (CPT1)	F	CTCTGCTGGCCGTTGTTGT	55	120
	R	GGCAAGTTCTGCCTCACGTA		
Sterol regulatory element-binding protein-2 (SREBP2)	F	CACAATATCATTGAAAAGCG	60	200
	R	TTTTTCTGATTGGCCAGCTT		
3-Hydroxy-3-methylglutaryl-CoA reductase (HMGCR)	F	TAAGATTCAACAACTCTGCT	55	101
	R	TGTGGCCAGGAGTTTGGTGA		
Farnesyl diphosphate synthase (FDPS)	F	ATGGAGATGGGCGAGTTCTT	60	80
	R	CCGACCTTTCCCGTCACA		
Cytochrome P450, family 51 (CYP51)	F	ACGCTGCCTGGCTATTGC	55	76
	R	TTGATCTCTCGATGGGCTCTATC		
Low-density lipoprotein receptor (LDLR)	F	AGGCTGTGGGCTCCATAGG	60	72
	R	TGCGGTCCAGGGTCATCT		
ATP-binding cassette, sub-family G, member 5 (ABCG5)	F	CGTGGCGGACCAAATGA	55	155
	R	CGCTCGCCACTGGAAATT		
ATP-binding cassette, sub-family G, member 8 (ABCG8)	F	TGCCCACCTTCCACATGTC	60	60
	R	ATGAAGCCGGCAGTAAGGTAGA		
Cytochrome P450, family 7, subfamily A, polypeptide 1 (CYP7A1)	F	CAGGGAGATGCTCTGTGTTCA	60	121
	R	AGGCATACATCCCTTCCGTGA		
Cytochrome P450, family 8, subfamily A, polypeptide 1 (CYP8B1)	F	AAGGCTGGCTTCCTGAGCTT	60	74
	R	AACAGCTCATCGGCCTCATC		
Acyl-coenzyme A: cholesterol acyltransferase (ACAT1)	F	AGCGAGACAGATGCTCATGC	55	107
	R	CAACCAAACCTCCGTCACTG		
Toll-like receptor 2 (TLR2)	F	GAGCATCCGAATTGCATCAC	55	120
	R	TATGGCCACCAAGATCCAGA		
Toll-like receptor 4 (TLR4)	F	TCGAATCCTGAGCAAACAGC	55	199
	R	CCCGGTAAGGTCCATGCTAT		
Toll-interleukin 1 receptor domain-containing adaptor protein (Tirap)	F	GCTTCCAGGGGATCTGATGT	55	183
	R	AAGCAAGCCTACCACGGACT		
TIR-domain-containing adapter-inducing interferon-*β* (TRIF)	F	ATGGATAACCCAGGGCCTT	55	528
	R	TTCTGGTCACTGCAGGGGAT		
Interferon regulatory factor 5 (IRF5)	F	ACCCGGATCTCAAAGACCAC	55	166
	R	TTATTGCATGCCAACTGGGT		
TNF alpha (TNF*α*)	F	TGTCTCAGCCTCTTCTCATT	55	156
	R	AGATGATCTGAGTGTGAGGG		
Interleukin 1 beta (IL-1*β*)	F	GTTGACGGACCCCAAAAGAT	55	129
	R	TGATACTGCCTGCCTGAAGC		
Interferon beta (IFN*β*)	F	TGGAGCAGCTGAATGGAAAG	55	122
	R	GAGCATCTCTTGGATGGCAA		
Glyceraldehyde-3-phosphate dehydrogenase (GAPDH)	F	CCCATGTTTGTGATGGGTGT	55	161
	R	GTGATGGCATGGACTGTGGT		
